# The Effects of Tetrapeptides Designed to Fit the Androgen Binding Site of ZIP9 on Myogenic and Osteogenic Cells

**DOI:** 10.3390/biology11010019

**Published:** 2021-12-23

**Authors:** Viveka Nand Malviya, Ahmed Bulldan, Raffael Christoph Wende, Hassan Kabbesh, Marie-Louise Möller, Peter Richard Schreiner, Georgios Scheiner-Bobis

**Affiliations:** 1 Max Planck Institute of Biophysical Chemistry, Institute of Neurobiology, Am Fassberg 11, 37077 Gottingen, Germany; viveka-nand.malviya@mpibpc.mpg.de; 2 Institute of Veterinary Physiology and Biochemistry, Justus Liebig University Giessen, Frankfurter Str. 100, 35392 Giessen, Germany; ahmed.bulldan@vetmed.uni-giessen.de (A.B.); hassan.kabbesh@vetmed.uni-giessen.de (H.K.); marie-louise.moeller@vetmed.uni-giessen.de (M.-L.M.); 3 Institute of Organic Chemistry, Justus Liebig University Giessen, Heinrich-Buff-Ring 17, 35392 Giessen, Germany; Raffael.Wende@org.Chemie.uni-giessen.de (R.C.W.); PRS@uni-giessen.de (P.R.S.)

**Keywords:** androgen receptor, mineralization, myogenic cells, myotube formation, osteogenic cells, testosterone-BSA-FITC, ZIP9

## Abstract

**Simple Summary:**

Pro-androgenic substances such as testosterone are often used to treat muscle- or bone-related disorders. Their interactions with the classical androgen receptor, however, can trigger a number of undesirable effects. It would therefore be of great benefit if the positive androgenic effects could be obtained by circumventing the classical androgen receptor. ZIP9 is a recently identified membrane-bound androgen receptor of physiological significance. Using in silico methods, we identified and verified the extracellular localization of its androgen binding site and designed small peptides that fit in it that do not interact with the AR. All peptides were found to be pro-androgenic; they stimulate mineralization in osteoblastic cells and myogenesis in myoblasts. Thus, these peptides might serve as testosterone surrogates in the treatment of osteogenic or myogenic disorders.

**Abstract:**

ZIP9 is a recently identified membrane-bound androgen receptor of physiological significance that may mediate certain physiological responses to androgens. Using in silico methods, six tetrapeptides with the best docking properties at the testosterone binding site of ZIP9 were synthesized and further investigated. All tetrapeptides displaced T-BSA-FITC, a membrane-impermeable testosterone analog, from the surface of mouse myogenic L6 cells that express ZIP9 but not the classical androgen receptor (AR). Silencing the expression of ZIP9 with siRNA prevented this labeling. All tetrapeptides were found to be pro-androgenic; in L6 cells they stimulated the expression of myogenin, triggered activation of focal adhesion kinase, and prompted the fusion of L6 myocytes to syncytial myotubes. In human osteoblastic SAOS-2 cells that express AR and ZIP9, they reduced the expression of alkaline phosphatase and stimulated mineralization. These latter effects were prevented by silencing ZIP9 expression, indicating that the osteoblast/osteocyte conversion is exclusively mediated through ZIP9. Our results demonstrate that the synthetic tetrapeptides, by acting as ZIP9-specific androgens, have the potential to replace testosterone or testosterone analogs in the treatment of bone- or muscle-related disorders by circumventing the undesirable effects mediated through the classical AR.

## 1. Introduction

The physiological effects of testosterone are mediated not only through the classical nuclear androgen receptors (AR), but also through membrane-bound receptors that trigger various cytosolic signaling cascades upon binding of the steroid hormone. One of these receptors is ZIP9, a zinc transporter from the family of ZRT/IRT-like proteins (ZRT = zinc-regulated transporter; IRT = iron-regulated transporter). ZIP9 mediates testosterone signaling events of physiological relevance through interactions with G-proteins [1,2,3]. It is known that in spermatogenic cells the non-classical signaling of testosterone, which involves the activation of a signaling cascade that contains the elements Erk1/2, CREB, and ATF-1 [4], is mediated through ZIP9. In Sertoli cells, testosterone/ZIP9 interactions stimulate the same signaling cascade leading to increased claudin expression and tight junction formation [5]. ZIP9-mediated signaling can be prevented by bicalutamide (Casodex) [6], an anti-androgen that is widely used in the clinic for the treatment of androgen-dependent malignancies [7]. Recent investigations also demonstrate a direct involvement of ZIP9 in the proliferation and migratory activity of the human metastatic prostate cancer cell line LNCaP [8].

All of these rather recent studies demonstrate the physiological relevance of ZIP9 and provide evidence that all the effects of testosterone described above are not due to the interactions of the androgen with the classical AR but result from its exclusive interactions with ZIP9. This conclusion is based partly on the fact that some of the cells used expressed ZIP9 but not the classical AR (e.g., 93RS2 Sertoli cells) [6]. In addition, all testosterone-induced effects described above disappear upon silencing the expression of ZIP9 by siRNA, whereas none of them are affected when the expression of the classical AR is restricted in an analogous way.

A first attempt to calculate a 3D model of ZIP9 resulted in a structure that resembled in many ways the classical zinc transporters with one significant difference: an extracellular cavity capable of accommodating and specifically interacting with testosterone or the anti-androgen bicalutamide [6]. The extracellular accessibility of the ZIP9 androgen binding site and the fact that its interactions with testosterone are of physiological significance bear the potential to specifically trigger ZIP9-specific testosterone effects, provided the existence of ZIP9-selective probes.

With this goal in mind, we re-calculated and further refined the model of the 3D structure of ZIP9 and identified by docking analysis several peptides that can specifically target the androgen binding site of the protein. For six of the most promising peptides (based on docking score, surface contact area, and atomic contact energy), we addressed their ability to displace T-BSA-FITC, a testosterone analog suitable for detecting plasma membrane-bound androgen receptors bearing an extracellularly accessible androgen binding site [6,9,10,11], and assessed their potential to induce testosterone-like effects in the rat-derived myogenic cell line L6 and the human-derived osteogenic cell line SAOS-2. Both cell lines are responsive to testosterone and react by either forming myotubes (L6) [12] or increasing mineralization (SAOS-2) [13]. They are therefore suitable to test the effects of the peptides and compare them with those of testosterone.

## 2. Materials and Methods

### 2.1. Molecular Modeling of ZIP9 and Docking Experiments to Identify Androgen Binding Sites

A homology model of ZIP9 (human) was generated by using the SWISS-MODEL server [14]. It utilizes the facility of an in-house comparative modeling engine ProMod3, based on OpenStructure [15]. The model building comprises four main stages: (a) structural template(s) identification from Protein Data Bank, (b) amino acid sequence alignment of template and target protein, (c) generation of a structural model for the target protein, (d) assessment of model quality. Initial structural information from the template structure was extracted by ProMod3, and final candidates were selected using potentials of mean force scoring methods. In our case, two structural templates were identified (PDB ID: 5tsa.1.A and 6pgi.1.A) from Bordetella bronchiseptica, which also belong to the ZRT/IRT-like protein family. The amino acid sequence of ZIP9 was obtained from the UniProt database (Q9NUM3). Both templates cover more than 90% of target sequence during sequence alignment and share 19.34% and 20.66% of sequence identity for 5tsa.1.A and 6pgi.1.A, respectively. Two models were generated using these two templates. To model non-conserved residues, an in-house rotamer library was used. A total of 291 and 292 amino acid residues out of 307 from ZIP9 were modeled using 5tsa.1.A and 6pgi.1.A templates, respectively. Another model was prepared using the user template mode of SWISS-MODEL server, and the mouse Zip9 structure [16] was used as a structure template. In total, 299 amino acid residues from both proteins were aligned and 93% of them were identical. Out of 307 residues from human ZIP9, 297 were modeled in the structure.

In the final step, energy minimization was performed using the CHARMM22/CMAP force field [6]. This was helpful for removing unfavorable bond interactions, distortion in bond angles, and other clashes. Finally, all models were analyzed using integrated model validation programs, and a comparison was made between two models (Table 1). From the table, it can be seen that the third model prepared using Zip9_mouse as a structure template was best among all created, so other computational analyses were performed using this model.

The simulation model was further refined manually by using COOT software [17]. Individual amino acid residues were checked for atomic clashes and correct rotamer. The final model obtained was subjected to a web-based protein structure validation server (PSVS) [18] that uses several other widely used structure validation tools like PROCHECK [19], MolProbity [20,21,22], Verify3D [23,24], ProSAII [25,26], and PDB validation software.

The patterns of nonbonded atomic interactions were analyzed by the ERRAT program. Various kinds of interactions between the protein and the ligand were visualized by BIOVIA Discovery Visualizer software (Dassault Systems BIOVIA, Discovery Studio Modeling Environment, Release 2017, San Diego, CA, USA: Dassault Systems, 2016). The final images were also prepared by using this software and PyMOL software (DeLano Scientific LLC, Version 2.4, https://pymol.org/2/, accessed on 23 December 2021).

### 2.2. Modeling Peptides within the Androgen Binding Site of ZIP9

Putative peptides that can bind within the same binding motif of the protein as bicalutamide and testosterone were designed by using the online server PepComposer [27], a tool that utilizes the protein structure coordinates and an approximate definition of the desired binding site on the protein. In this case the PDB coordinates of the model structure of human ZIP9 protein were provided along with the few amino acid residues present in the binding pocket. The method is based on the derivation of a set of peptide backbone scaffolds from monomeric proteins that harbor the same backbone arrangement as the binding site of the protein of interest and retrieve putatively interacting peptide backbones from them. Next, it uses Monte Carlo to design optimal sequences for the peptide scaffolds identified. The average FoldX binding energy (kcal/mol) of the individual peptide and protein complex is generated by FoldX server [28].

Candidate peptides were further verified independently by the docking server PatchDock [29,30] by extracting the PDB coordinates of each peptide from the complex and subjecting it to docking using the ZIP9 model coordinates as the receptor molecule. The AutoDock Vina program [31] was used to generate ZIP9–peptide complexes. AutoDock Vina uses an explicit coordinate-defined rectangular box as a search space on the receptor molecule for determining the ligand binding conformation. Since the center of the box and the size can be adjusted to the user’s demand, in this case, a relatively large rectangular box was created that covered the testosterone binding site of the protein for which these peptides were generated. OpenBabel 2.2.3 (https://sourceforge.net/projects/openbabel/files/openbabel/2.2.3/, accessed on 23 December 2021) was used to assign the hydrogen atoms to the ligand molecule [32]. Both receptor and ligand molecules were represented in a modified PDB format called pdbqt created by AutoDockTools 1.5.6 (Molecular Graphics Laboratory, The Scripps Research Institute, La Jolla, CA, USA). Using this tool, hydrogen atoms were added to the polar residues of the protein molecule. The PDBQT format contains information about the atom type and surface charges on protein molecule as well as rotational bonds present in the ligand molecule. The torsion angle of all rotational bonds of the ligand were set to random, and the translation and orientation of the ligand was also random. After the input files were ready, AutoDock Vina wizard was run through PyRx-Virtual Screening Tools (Molecular Graphics Laboratory, The Scripps Research Institute, La Jolla, CA, USA).

### 2.3. Cell Culture of Rat-Derived Myoblast Cells

Immortalized L6 myoblast cells (ATCC, Manassas, VA, USA) derived from rat quadriceps skeletal muscle [33] were seeded at a density of 2 × 10^4^ per well in DMEM-12 complete growth medium containing 10% fetal bovine serum (FBS) (Biochrom, Berlin, Germany), 1% L-glutamine (Gibco, Darmstadt, Germany), and 1% penicillin/streptomycin (Gibco). Cells were cultured in a humidified incubator at 37 °C under 5% CO_2_. The cell medium was replaced every two days. Cells were passaged up to 10 times.

### 2.4. Cell Culture of Human-Derived Osteogenic Cells

SAOS-2 osteogenic cells (DSMZ, Braunschweig, Germany) derived from primary osteosarcoma of an 11-year-old Caucasian girl [34] were cultured at a density of 3 × 10^4^ per well in McCoy’s 5 A medium (Gibco, Darmstadt, Germany) containing 10% FBS, 1% L-glutamine, and 1% penicillin/streptomycin. Cells were cultured in a humidified incubator at 37 °C under 5% CO_2_. Growth medium was replaced every two days. Cells were passaged up to 10 times.

### 2.5. Cell-Surface Labeling with Testosterone-BSA-FITC

L6 myogenic cells were cultured as described above in 24-well plates at a density of 5 × 10^3^ cells per well. After reaching a confluence of approximately 80%, the cells were treated with either 10 nM testosterone or 1 µM of each of the synthetic peptides for 1 h. The ethanol vehicle for testosterone was added to the peptide-treated cells and untreated controls at the same concentration as the present cell cultures treated with testosterone. Thereafter, testosterone 3-(O-carboxymethyl)oxime:bovine serum albumin-fluorescein isothiocyanate (T-BSA-FITC) conjugate (Sigma-Aldrich, Munich, Germany) diluted in Tris buffer (pH 7.2) was added to each well at a final concentration of 5 µM, and incubation was continued at room temperature for another 20 min. The medium was then removed by aspiration. In order to label nuclei, cells were fixed at room temperature with 3.7% formaldehyde that contained 20 ng of 4,6-diamino-2-phenylindole (DAPI). After 15 min, the formaldehyde/DAPI solution was removed by aspiration. Cells were washed with 700 μL phosphate-buffered saline (PBS) and then overlayed with 400 µL PBS before imaging. Images were taken by an inverse Olympus IX81 microscope (Olympus, Hamburg, Germany).

Non-specific binding was assessed by incubating the cells with 5 µM BSA-FITC (lacking testosterone) diluted in Tris buffer (pH 7.2) for 20 min at room temperature. Fixation and imaging were carried out as described in the previous paragraph.

### 2.6. Preparation of Cell Lysates of L6 Myoblasts

A total of 2 × 10^4^ L6 cells per dish were grown in 5-cm culture dishes as described above. Cells were then incubated for 24 h with 1% FBS before synthetic peptide-1 or peptide-2 (Pep-1, Pep-2) were added to the medium to reach the desired final concentration. The ethanol vehicle for testosterone was added to the peptide-treated cells and untreated controls at the same concentration as the present cell cultures treated with testosterone. At the end of the desired incubation time, the medium was aspired and the cells subsequently washed in ice-cold PBS (without Ca^2+^ or Mg^2+^; Thermo Fisher Scientific, Darmstadt, Germany) twice. In order to obtain lysates, cells were incubated with 400 μL of a commercially available lysis buffer (Cell Signaling Technology, Frankfurt, Germany), containing 1 μM PMSF, 1× protease inhibitor cocktail (Roche, Mannheim, Germany), and 2 μg per mL-1 pepstatin that was added immediately before use. All further steps were carried out on ice. After 5 min of incubation, cells were detached from the dish with a cell scraper. The suspensions were then transferred into 1.5 mL reaction vials and sonicated 5 times for 1 s each with intervals of 1 s. After centrifugation of the lysates at 4 °C and 13,000× *g* for 10 min, the protein concentration in the supernatants was determined at 540 nm using the bicinchoninic acid protein assay reagent kit (Pierce, Southfield, MI, USA) and a Labsystems (Helsinki, Finland) plate reader. The BSA protein standard contained lysis buffer at the same concentration as in the samples from the cell lysates. Supernatants were then aliquoted and maintained at −20 °C until further use.

### 2.7. Western Blotting

A total of 10 μg protein from L6 cell lysates were run on SDS-PAGE gels containing 10% acrylamide and 0.3% N,N′-methylenebis-acrylamide. As molecular weight markers, biotinylated proteins (Cell Signaling Technologies, Frankfurt, Germany) were used that were run in parallel. After electrophoresis, proteins were electro-blotted onto PVDF membranes (Merck Chemicals GmbH, Schwalbach, Germany) for 30 min at 0.5 V cm^−2^. The membranes were then incubated for 1 h at room temperature in 5% non-fat milk. Appropriate primary antibodies against myogenin or beta-actin were diluted according to the recommendations of the providers and then incubated with the membranes overnight at 4 °C. After washing in PBS, the membranes were incubated for 60 min at room temperature with the horseradish peroxidase-conjugated secondary antibody. An anti-biotin, HRP-conjugated antibody (Cell Signaling Technologies) at a dilution of 1:2000 was also included in the mixture containing the secondary antibody in order to detect the biotinylated molecular weight marker. Antibody-labelled protein bands were visualized by enhanced chemiluminescence [35].

### 2.8. Immunofluorescence in Myogenic Cells

The L6 myoblasts were cultured as described above in 24-well culture plates. To induce L6 myoblast differentiation into myocytes and myotubes, cells were switched to the same DMEM medium containing 1% FBS. After 24 h, each sample received one of the following treatments: 10 nM testosterone, 10 µM Pep-1, 10 µM Pep-2, or vehicle, which served as control. The incubations were stopped after various times by fixing the cells with either ice-cold methanol or formaldehyde 3.7%, depending on the type and nature of the parameter under investigation. After 15 min of incubation at room temperature, the fixative was aspirated and the cells were rinsed with PBS. Thereafter, the cells were blocked with 3% BSA and 0.3% Triton-X100 in PBS for 1 h at room temperature. The first antibody against focal adhesion kinase p-FAK (Tyr925) or myogenin was added according to the recommendation of the provider, and incubation was continued overnight at 4 °C. Staining was accomplished by adding AlexaFluor-labeled secondary antibody for 1 h at room temperature (see below).

### 2.9. Immunofluorescence in Osteogenic Cells

A total of 1 × 10^5^/well SAOS-2 cells were added into a 24-well culture plate. To investigate how testosterone or Pep-1 might influence alkaline phosphatase staining, the cells were incubated for 48 h either with testosterone (10 nM), Pep-1 (10 µM), or vehicle. The medium was then aspirated and the cells were fixed with absolute methanol containing DAPI (4′,6-diamidino-2-phenylindole) for 10 min at room temperature. Then the solution was aspirated and the samples were washed 3 times in 500 μL PBS. The cells were then blocked with 3% BSA and 0.3% Triton-X100 in PBS for 1 h at room temperature. Thereafter, the cells were incubated with 1% BSA and 0.1% Triton-X100 in PBS containing antibodies against alkaline phosphatase for 1 h at room temperature. After the incubation step with the primary antibody, the plate was washed 3 times for 5 min with 500 μL PBS per well. Staining was accomplished by adding AlexaFluor-labeled secondary antibody for 1 h at room temperature (see next paragraph).

### 2.10. Fluorescence Staining, Recording, and Quantification

For fluorescence staining of myogenin or p-FAK in myogenic L6 cells or alkaline phosphatase in osteogenic SAOS-2 cells, the medium containing the primary antibody was replaced with PBS containing 1% BSA, 0.3% Triton X-100, and the secondary antibody (goat anti-rabbit IgG diluted at 1:500 in 2% FCS, 0.1 Triton X100 in PBS) labeled with AlexaFluor 488 (Life Technologies, Darmstadt, Germany) was added for 1 h at room temperature. The incubation medium was then aspirated and cells were washed 3 times in PBS. All incubation steps with UV-sensitive reagents were carried out in the dark. The images were acquired by an inverse Olympus IX81 microscope equipped with the corresponding fluorescence system (Olympus, Hamburg, Germany).

Fluorescence was measured using the software program ImageJ (freely available at http://rsbweb.nih.gov/ij/, accessed on 23 December 2021) according to the protocol provided (http://www.slu.se/PageFiles/388774/Pacho%20ImageJ%20-measuring-cell-fluorescence.pdf, accessed on 23 December 2021). For all quantification or for evaluation of myogenin expression, only green fluorescence was considered. For statistical analysis of myogenin expression, all cells in the optical field were considered. Data points were transferred to and analyzed by the software program GraphPad Prism4 (GraphPad Software, Inc., La Jolla, CA, USA).

### 2.11. Detection of Actin Fibers in L6 Myoblasts

Cells were incubated for 9 h with testosterone or Pep-1 as described above. Controls received vehicle only. To evaluate and visualize the L6 myoblast, cell alignment, and cell fusion to myotubes, the L6 cytoskeleton F-actin was stained with phalloidin (Sigma-Aldrich, Steinheim, Germany). The phalloidin staining was achieved by diluting the phalloidin in incubation media at the concentration recommended by the provider overnight at 4 °C in a humidified chamber. Nuclear staining was obtained by adding 20 ng of DAPI diluted in PBS for 10 min. Images were obtained using an Olympus inverse microscope (Olympus, Hamburg, Germany) over random areas of the field at 10 × magnification.

### 2.12. Alizarin Red Staining and Quantification

SAOS-2 osteoblast cells (2 × 10^5^ cells/well) were cultured in 24-well culture plates. The McCoy’s 5 A osteogenic medium was further supplemented with ascorbic acid 0.1 M (Sigma-Aldrich), glycerophosphate 1 M (Sigma-Aldrich), and dexamethasone 1 mM. To investigate the extent to which the testosterone or peptides induce osteoblast mineralization and differentiation, the cells were treated with either 10 nM testosterone, 10 µM Pep-1, or vehicle, which served as a control. For the long-term experiment addressing the effect of the various treatments, the osteogenic medium was carefully replaced every 2 days with fresh medium containing the appropriate concentration of testosterone, Pep-1, or vehicle. The experiment was stopped at 0, 4, and 11 days by fixing the cells with 3.7% formaldehyde for 30 min at 4 °C. Mineralization and calcium deposition were assessed by staining the cells with Alizarin Red S sodium salt (3,4-dihydroxy-9,10-dioxo-2-anthracenesulfonic acid sodium salt) (Alfa Aesar, Kandel, Germany) dissolved in distilled water (pH 4.2 + solution filtration) for 1 h in the dark and under shaking. Images of stained surfaces were acquired at the previously mentioned time points of incubation using a light microscope (Olympus CK2 Inverted Binuclear, Hamburg, Germany).

For quantification of mineralization and calcium deposition, the Alizarin Red S-stained SAOS-2 cells were washed 3 times with distilled water. The Alizarin staining was extracted using 10% hexadecyl pyridinium chloride monohydrate (Sigma-Aldrich) dissolved in distilled water. After a 2-h incubation, the extract was transferred into a 96-well plate and the absorbance was measured at 540 nm in triplicate using a plate reader (Lab Systems, Helsinki, Finland).

### 2.13. Silencing ZIP9 Expression

In order to investigate the involvement of ZIP9 and reveal a possible participation of the classical AR in the peptide-induced effects, ZIP9 expression in human SAOS-2 cells was suppressed using siRNA as described earlier for human LNCaP prostate cancer cells [8]. The two oligonucleotides used for silencing ZIP9 expression in human SAOS cells were 5′GGCUUAGAGCGGAAUCGAAtt3′ and 5′UUCGAUUCCGCUCUAAGCCag3′. Likewise, ZIP9 expression in rat-derived L6 myogenic cells was achieved as described earlier for the suppression of ZIP9 expression in rat-derived 93RS2 Sertoli cells [5]. The two oligonucleotides used for silencing ZIP expression in rat cells were 5′ GGAUUAAGUAAGAGCAGUAtt3′ and 5′ UACUGCUCUUACUUAAUCCta 3′. All oligonucleotides were purchased from Thermo Fisher Scientific, Darmstadt, Germany. Negative control siRNA (nc-siRNA) was included in the siRNA kit provided by the same manufacturer.

### 2.14. Statistical Analysis

Data were analyzed by GraphPad Prism4 software and by applying one-way ANOVA with repeated measures and Dunnett’s comparison of all data to the control. Significance was accepted at *p* < 0.05.

## 3. Results

### 3.1. Identification of the Putative Androgen Binding Site of ZIP9 by In Silico Calculations

Three models were generated for ZIP9 protein using the SWISS-MODEL server and a structural comparison was made (Table 1). Since the third model had the lowest clash score and MolProbity score, it was considered to be the best and was further used for other computational analyses. A low MolProbity score and a negligible clash score indicates a highly reliable model. The simulated model was further refined manually on COOT. Further corroboration of the final structure by PDB validation software did not reveal any close contacts in the model, and RMSD for bond angles and bond lengths were 2.4° and 0.016 Å, respectively. The φ/ψ angle distribution was also within allowed limits. Further analysis by the ProCheck program confirmed the presence of 81.4% of the residues in the most-favored regions, and in the Ramachandran plot 97.0% of the residues were identified within allowed regions. The mean value of the MolProbity score was 3.82, which represents a highly accurate model with very few atomic clashes within the molecule. The Z-score of the model calculated by ProSAII was −3.8, which is in the range of the Z-score of all experimentally determined protein chains present in PDB and therefore represents a reliable native conformation of the protein. A total of 80% of the amino acids have scored ≥0.2 in the 3D/1D profile generated by Verify3D. The overall quality factor of 91% provided by the ERRAT server also suggests that the model is highly accurate.

The predicted 3D structure of ZIP9 at expected locations bears typical characteristics of zinc transporters [6,36,37,38] and supports the previous model of ZIP9 that had been generated by different modeling methods [6]. In contrast to the other members of the ZRT/IRT family of proteins, however, the surface electrostatic potential map of the ZIP9 protein shows a large binding pocket present in the extracellular region very close to the lipid bilayer membrane. The identified binding pocket corresponds to the testosterone and bicalutamide binding site that had been identified earlier by a different modeling approach [6]. This region of the protein is lined by amino acids carrying nonpolar side chains and is therefore suitable for interacting with hydrophobic peptide molecules. The volume of the pocket is large enough to accommodate tri- or tetrapeptides.

### 3.2. Identification of Peptides Fitting the Androgen Binding Site of ZIP9

The PepComposer server generated many peptide sequences with a high propensity for binding to the protein molecule in the same binding pocket as testosterone and bicalutamide. From the various peptides generated, there were only six complexes found to have negative FoldX energy (Table 2), which is an indication of a highly stable protein–peptide interaction.

**Figure 1:**
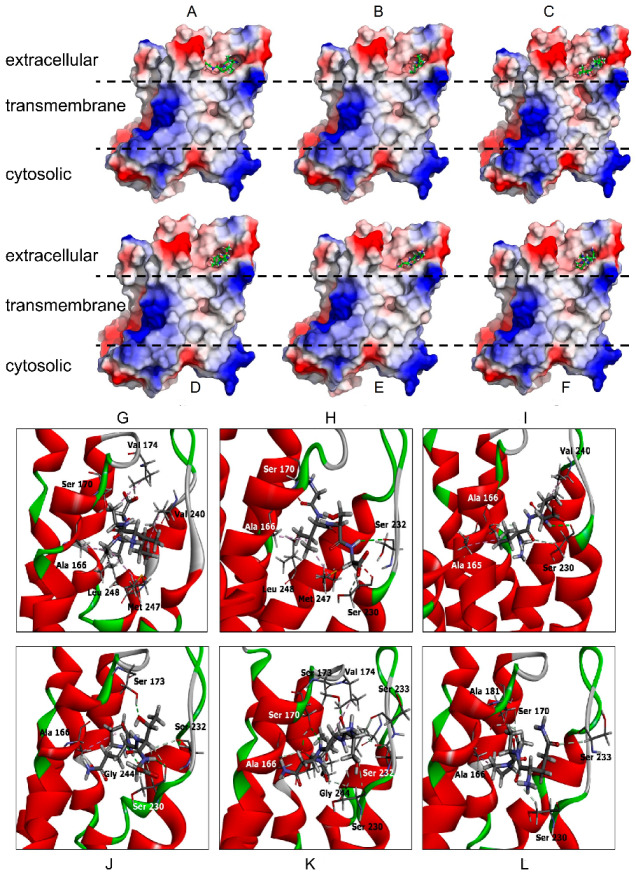
ZIP9-peptide complex model obtained via PepComposer and PatchDoc server. (**A**–**F**) Complexes formed between ZIP9 and peptides IAPG, GVSG, GVVG, PQTG, SGNL, and QAPG, respectively. All of them bind within the predicted extracellular androgen binding site of the protein. (**G**–**L**) ZIP9–peptide complex models showing molecular details of interactions of all amino acid residues from the androgen binding site whose side chains are involved in direct interactions with the peptides IAPG, GVSG, GVVG, PQTG, SGNL, and QAPG, respectively.

All six complexes were further verified by a different docking server, PatchDock [29,30]. The PDB coordinate of each peptide was extracted from the complex and subjected to docking via the PatchDock server using ZIP9 model coordinates as the receptor molecule. Many protein–peptide complexes were generated, and the complex that had highest docking score, maximum surface contact area, and minimum atomic contact energy was selected. The first three peptides (IAPG, GVSG, and GVVG) in complex with the ZIP9 protein had the same order of docking score, surface contact area, and atomic contact energy obtained from PatchDock as their negative FoldX energy values obtained via PepComposer (Table 2). Therefore, these two independent calculations yielded results in good agreement with each other, further enhancing the reliability of the modeled complexes. The detailed analysis of the complexes showed that these peptides bind within the same binding pocket at the extracellular surface of the protein (Figure 1). This is the same binding site for testosterone that was identified earlier [6]. The docked peptides interact with the side chains of several amino acids (Figure 1) that were shown earlier to also interact with testosterone [6]. Table 3 shows a synopsis of all amino acids involved in direct interactions with either testosterone or any of the peptides. Only amino acid A166 is involved in direct interactions with all the compounds.

The results obtained from both methods demonstrate with a high degree of confidence that the six peptides selected are highly likely to bind in the same binding pocket as testosterone. The atomic contact energy of these peptides is similar to that of testosterone [6]. Based on these results, these six peptides were selected as potential candidates for synthesis and for the screening of their properties.

All peptides were also docked on the ZIP9 model protein using the AutoDock Vina program. The Vina program generated eight protein models docked with different peptides in different conformations. These models were ranked according to the binding energy between protein and peptides. The protein–peptide complex having the highest binding affinity was selected for further analysis. Such complexes were generated for all six peptides individually. A detailed analysis of the molecular interactions between protein and each peptide molecule was conducted using Discovery Visualizer software (Dassault Systems BIOVIA, Discovery Studio Modeling Environment, Release 2016, San Diego, CA, USA: Dassault Systems, 2016). Peptides were ranked according to their binding energy (kcal mol^−1^) as listed in Table 2.

### 3.3. Synthesis, Analysis, and Purification of Tetrapeptides

Tetrapeptides PQTG, IAPG, QAPG, GVSG, GVVG, and SGNL were synthesized by 1-(3-dimethylaminopropyl)-3-ethylcarbodiimide hydro-chloride (EDC·HCl) and 1-hydroxybenzotriazole hydrate (HOBt·H_2_O)-mediated coupling in solution using the N-tert-butoxycarbonyl (Boc) protected amino acids. Benzyl ester, benzyl-oxycarbonyl (Cbz) and benzyl ether protecting groups were employed for the C-terminal and N-terminal amino acids, and the side chains of serine and threonine, respectively. The final deprotection step was achieved by hydrogenation with palladium on charcoal. A representative synthesis is shown in Supplementary Figure S1. The crude peptides were subsequently purified by reversed-phase high-performance liquid chromatography (RP-HPLC), lyophilized, and characterized by nuclear magnetic resonance (NMR) spectroscopy and high-resolution mass spectrometry (HRMS). The purity of the final products was determined by analytical HPLC and was found to be >95% for PQTG, IAPG, QAPG, GVSG, and SGNL and 95% for GVVG. The detailed syntheses, analytical data, as well as chromatograms, can be provided upon request.

### 3.4. Binding of Peptides to the Androgen Binding Site of ZIP9

**Figure 2:**
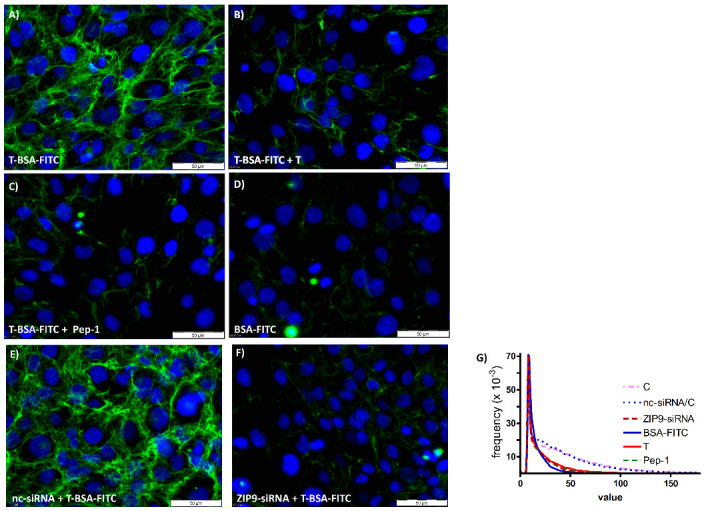
Displacement of T-BSA-FITC from the extracellular androgen binding site of L6 myoblasts by the synthesized peptides and testosterone. Nuclei are stained blue with DAPI; fluorescence corresponding to T-BSA-FITC is green. (**A**) When control cells are incubated with T-BSA-FITC, the compound labels their plasma membranes. (**B**) The simultaneous presence of testosterone (T) or (**C**) IAPG (Pep-1) greatly prevents labeling with T-BSA-FITC. The remaining labeling with green fluorescence does not differ significantly from the labeling obtained with BSA-FITC (**D**), indicating that it is due to non-specific interactions of either of the two compounds with some cellular components. (**E**) When cells that had been treated with negative control siRNA (nc-siRNA) are incubated with T-BSA-FITC, the testosterone analog labels their membranes as in untreated controls shown in (**A**). (**F**) Silencing ZIP9 expression by ZIP9-specific siRNA (ZIP9-siRNA) entirely prevents binding of T-BSA-FITC, indicating that in these cells no other membrane-bound protein capable of specifically binding testosterone exists besides ZIP9. (**G**) Plots of green fluorescence intensity versus frequency summarize and support the conclusions listed above; treatment with nc-siRNA does not affect the binding of T-BSA-FITC, since this and the curve obtained under control conditions are almost identical. It also shows that testosterone and Pep-1 reduce the frequency of strong fluorescent signals to about the same extent. Similar results are obtained with BSA-FITC or after silencing ZIP9 expression by siRNA.

T-BSA-FITC is a testosterone analog that cannot penetrate the plasma membrane [6,9,10,11]. It is therefore suitable to detect plasma membrane-bound androgen receptors bearing an extracellularly accessible androgen binding site. Incubation of L6 myogenic cells with T-BSA-FITC led to the uniform green labeling of the extracellular surface of the cells, supporting the calculated 3D model of ZIP9 that identifies its androgen binding site on its extracellular surface (Figure 2A). When testosterone is included in the incubation medium (Figure 2B) or either of the peptides (Figure 2C; shown in a representative way for Pep-1), membrane labeling by T-BSA-FITC is greatly suppressed. Residual, relatively low levels of green fluorescence are also obtained when cells are incubated with BSA-FITC (lacking the testosterone moiety; Figure 2D), indicating that this residual labeling is due to non-specific interactions of the fluorescent compounds with some components of the plasma membrane or the cytosol.

To ensure that testosterone-BSA-FITC or the peptides specifically target the ZIP9 protein and not some other components that might also bear nonspecific androgen binding sites, labeling experiments were repeated after silencing the expression of ZIP9 by siRNA. Figure 2E shows that the treatment of the cells with negative control siRNA (nc-siRNA) did not affect the labeling of the cell membranes by T-BSA-FITC. When, however, the expression of ZIP9 was suppressed by ZIP9-specific siRNA (ZIP9-siRNA), labeling of the cell membranes was completely prevented and only some cytosolic, non-specific labeling was seen in a few cells (Figure 2F).

A plot of fluorescence intensity versus frequency (Figure 2G) shows an almost complete overlap of the lines obtained for the two controls (without or with nc-siRNA), indicating that the treatment with nc-siRNA did not affect the expression of ZIP9 and thus the binding of T-BSA-FITC. Incubation with testosterone or Pep-1 suppressed strong fluorescent signals to about the same extent, shifting the highest frequency to lower values of fluorescence (the other peptides produce similar effects; not shown). A similar reduction in signals was also observed when ZIP9 expression was prevented by siRNA.

### 3.5. Physiologically Relevant Responses to Peptides in Myogenic Cells

**Figure 3:**
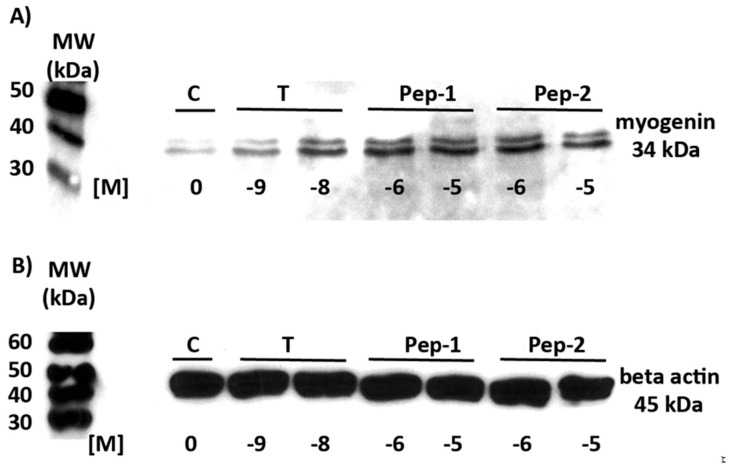

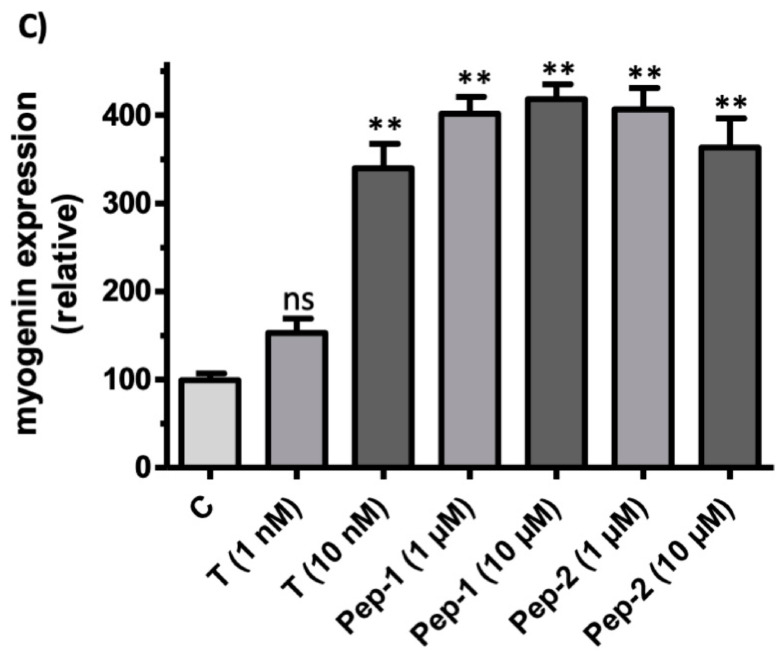
Detection of myogenin in western blots. Cell lysates were prepared from untreated L6 cells or from cells that had been exposed to different concentrations of testosterone, Pep-1, or Pep-2. (**A**) All androgenic compounds stimulate expression of myogenin. (**B**) The expression of beta-actin is not affected under any of the conditions applied. (**C**) Quantitative analysis of results as shown in (**A**). Data were corrected for (normalized to) the amount of beta-actin as shown in (**B**) (*n* = 3; means ± SEM; ** *p* ≤ 0.001).

Myogenin and focal adhesion kinase (FAK) are key players of skeletal myogenesis and are regulated by testosterone [39,40,41,42,43]. It is therefore important to address the effects of the peptides and testosterone on these two proteins. The effects of the peptides on myogenin expression were determined and compared with the effects of testosterone. Western blotting for myogenin was carried out in lysates from cells that had been incubated with various concentrations of testosterone, Pep-1, or Pep-2. Within 6 h, all peptides and testosterone stimulated the expression of myogenin (Figure 3A and Figure S2), which was not due to a general stimulation of protein expression, as demonstrated by the lack of an effect on beta-actin expression (Figure 3B and Figure S2).

**Figure 4:**
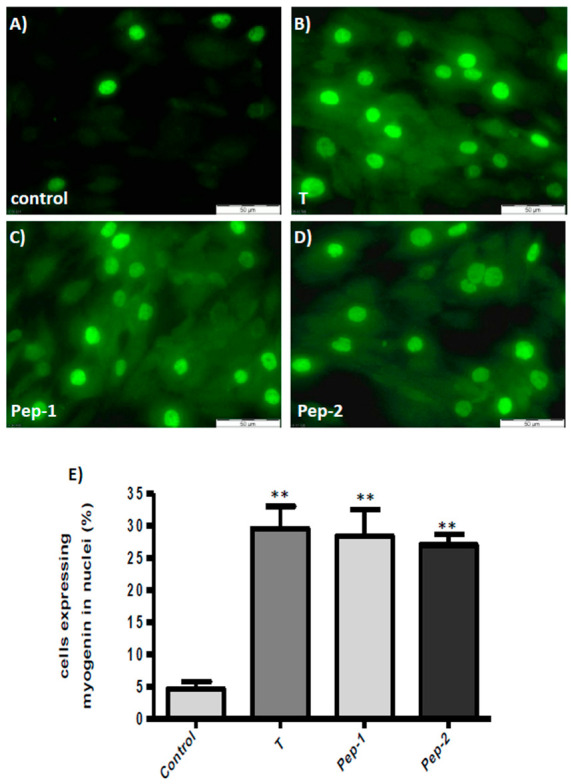
Detection of myogenin in L6 cells by immunofluorescence. DAPI blue staining of nuclei is not shown in order to avoid interference with the green fluorescence staining of myogenin. (**A**) Detection of myogenin in control cells. (**B**) Detection of myogenin in nuclei and cytosol of cells treated with testosterone. (**C**) Detection of myogenin in nuclei and cytosol of cells treated with Pep-1. (**D**) Detection of myogenin in nuclei and cytosol of cells treated with Pep-2. (**E**) Quantitative analysis showing the number of green-stained nuclei as % of the total nuclei present in the optical field. While not all nuclei were stained green, green fluorescence was visible within the cytosol of each individual cell that had been exposed to testosterone, Pep-1, or Pep-2, indicating stimulation of its expression by the three androgenic compounds. (*n* = 3 ± SEM; ** *p* ≤ 0.001).

The response of individual cells to the stimulus and the localization of myogenin was addressed by immunofluorescence. In the absence of any stimulus a few cells (5 ± 1.5%) displayed myogenin within their nuclei (Figure 4A,E), as expected for transcription factors. There was no visible green fluorescence within the cytoplasm of the cells, indicating that there was no stimulation of myogenin expression under these conditions. The addition of testosterone or any of the peptides stimulated the expression of myogenin to about the same level. Thus, in the presence of testosterone, Pep-1, or Pep-2, myogenin was visible in 29 ± 4%, 28 ± 3%, or 27 ± 2% of the L6 myogenic cell nuclei, respectively (Figure 4B–E). In contrast to the untreated controls, green fluorescence is also seen within the cytosol of each individual cell in cultures treated with testosterone, Pep-1, or Pep-2, consistent with the stimulation of myogenin biosynthesis at ribosomes by these androgenic compounds.

**Figure 5:**
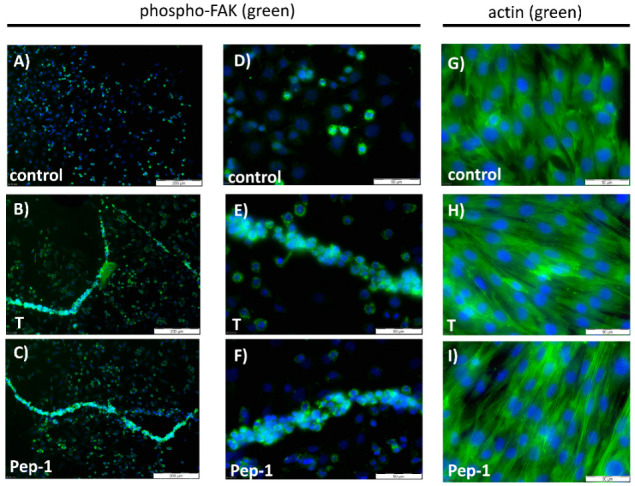
Demonstration of syncytial myotube formation induced by testosterone or peptides. All photomicrographs in (**A**–**I**) are taken after 9 h of incubation. Nuclei are stained with DAPI (blue). (**A**–**F**): Green fluorescence corresponds to FAK phosphorylated at Y925. (**A**) Untreated control cells. (**B**) Treatment with testosterone (T) stimulates both FAK phosphorylation and induces the formation of myotubes. (**C**) Pep-1 also stimulates FAK phosphorylation and myotube formation. (**D**–**F**) The higher magnification reveals single-cell structures and the focal localization of phospho-FAK. (**G**–**I**) Green fluorescence corresponds to actin filaments labelled with phalloidin. (**G**) Detection of phalloidin-stained actin filaments in control cells. The actin filaments are contained in each individual cell and cells are randomly oriented. (**H**) Cells treated with testosterone form parallel-oriented syncytia with actin fibers stretching over several cells. (**I**) A similar pattern as that shown in (**H**) is obtained when cells are treated with Pep-1.

Figure 5 shows the effects of testosterone or Pep-1 on the activity of FAK (phosphorylation at Tyr925) in L6 myogenic cells and on the formation of myotubes by these myogenic cells after 9 h of incubation. When compared with untreated controls, almost all cells treated with testosterone or Pep-1 showed p-FAK-specific green fluorescent staining (Figure 5B,C), whereas less than 15% of the untreated control cells displayed a signal (Figure 5A). Similar results concerning the phosphorylation status of FAK were obtained after 3 or 6 h of incubation (not shown). A major difference compared with the earlier time points, however, is that after 9 h cells treated with testosterone or Pep-1 started to assemble into myotubes (Figure 5B,C). Similar structures were not seen in the absence of androgenic activity (Figure 5A). At higher magnifications (Figure 5D–F), the single-cell structures and the foci containing p-FAK localized therein were readily apparent.

The results displayed in Figure 5A–F showing myotube formation were confirmed by the phalloidin staining of actin filaments of the L6 cells. As can be seen in Figure 5H,I, actin fibers stretching over several aligned nuclei were observed in the presence of testosterone or Pep-1, indicating the formation multinucleated syncytial myotubes. Similar structures were lacking in the absence of androgenic stimuli (Figure 5G).

### 3.6. Physiologically Relevant Responses in Osteogenic Cells

Expression levels of bone-specific alkaline phosphatase are closely and reversibly associated with circulating testosterone concentrations and bone mineralization [44,45]. It is therefore important to assess the effects of testosterone and the peptides on alkaline phosphatase expression and mineralization in human osteogenic SAOS-2 cells.

**Figure 6:**
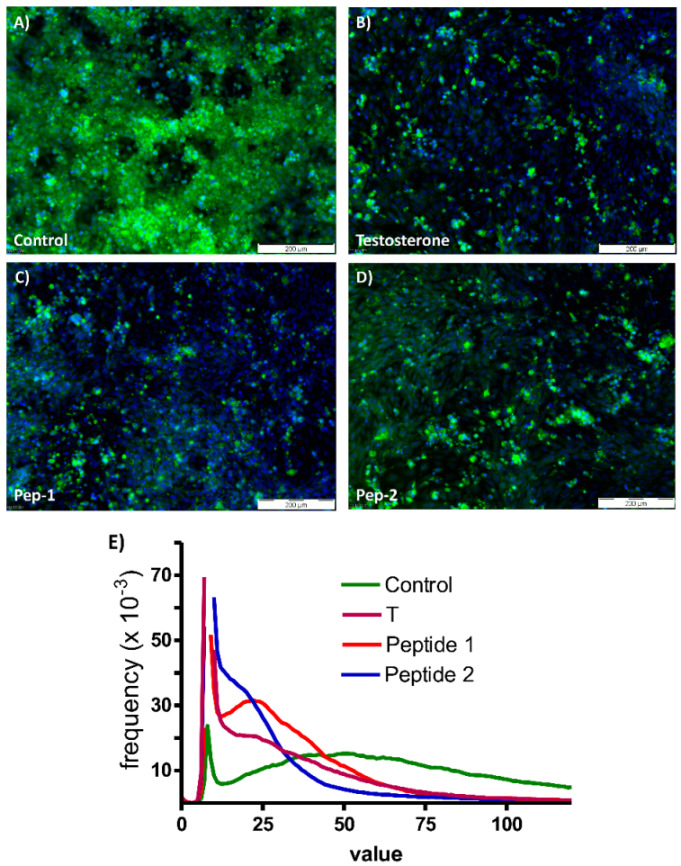
Detection of alkaline phosphatase in SAOS-2 cells by immunofluorescence. In all frames alkaline phosphatase is indicated by the green fluorescence and nuclei are stained blue by DAPI. (**A**) Expression of alkaline phosphatase in SAOS-2 cells in the absence of testosterone or peptides. (**B**) Expression of alkaline phosphatase in the presence of testosterone. (**C**,**D**) Expression of the same enzyme in the presence of Pep-1 or Pep-2, respectively. (**E**) Plots of green fluorescence intensity versus frequency obtained in the presence of testosterone, Pep-1, or Pep-2 are highly divergent from that of the control.

Figure 6 shows the expression of bone-specific alkaline phosphatase after 48 h of incubation with vehicle (A), testosterone (B), Pep-1 (C), or with Pep-2 (D). Testosterone and both peptides significantly reduced the expression of alkaline phosphatase in the osteogenic SAOS-2 cells (Figure 6E).

Testosterone and both Pep-1 and Pep-2 were also found to stimulate mineralization and calcium deposits in SAOS-2 osteogenic cells. As shown in Supplementary Figure S3, positive Alizarin Red staining was already clearly visible four days after the incubation of the cells with testosterone or the androgenic peptides (Supplementary Figure S3B–D), whereas control cells displayed only very modest mineralization under these conditions (Supplementary Figure S3A). After 11 days, cells in the optical field that had been treated with testosterone or peptides (Supplementary Figure S3F–H) showed markedly increased amounts of calcium deposits compared with that observed in control cells (Supplementary Figure S3E).

### 3.7. Role of ZIP9 in Mineralization Process Induced by Testosterone and Peptides in Osteogenic Cells

**Figure 7:**
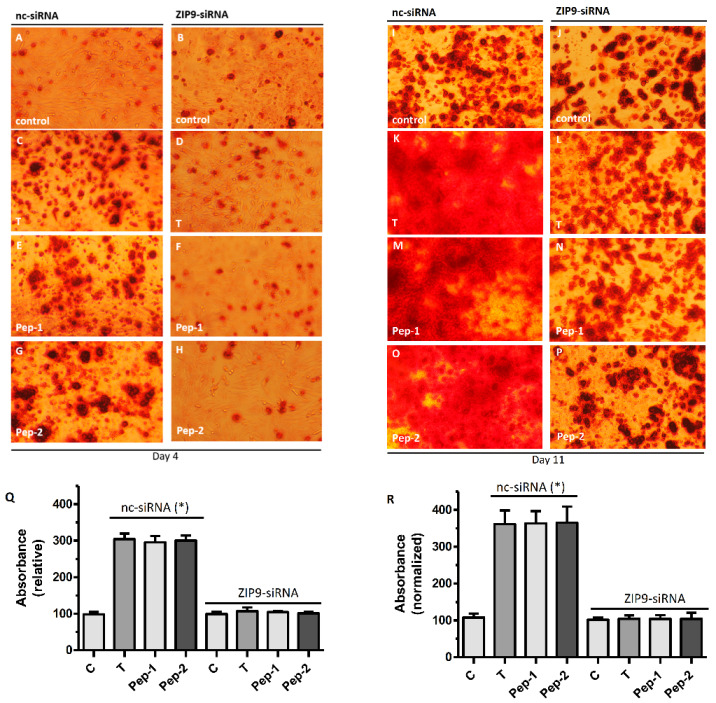
Alizarin Red staining of osteoblastic SAOS-2 cells after silencing ZIP9 expression. Cells were treated with either negative control siRNA (nc-siRNA; all left-hand panels) or ZIP-specific siRNA (ZIP9-siRNA; all right-hand panels) to silence ZIP9 expression. Alizarin Red staining was performed after 4 (**A**–**H**) or 11 (**I**–**P**) days of incubation with testosterone, Pep-1, or Pep-2. Controls received vehicle only. After 4 days of incubation: (**A**,**B**) Alizarin Red staining in control cells; (**C,D**) Alizarin Red staining in the presence of testosterone; (**E**,**F**) Alizarin Red staining in the presence of Pep-1; (**G**,**H**) Alizarin Red staining in the presence of Pep-2. After 11 days of incubation: (**I**,**J**) Alizarin Red staining in control cells; (**K**,**L**) Alizarin Red staining in cells treated with testosterone; (**M**,**N**) Alizarin Red staining in cells treated with Pep-1; (**O**,**P**) Alizarin Red staining in cells treated with Pep-2. (**Q**) Quantification of Alizarin Red after 4 days of incubation (*n* = 3; means ± SEM; * *p* ≤ 0.05). (**R**) Quantification of Alizarin Red after 11 days of incubation (*n* = 3; means ± SEM; * *p* ≤ 0.05).

SAOS-2 cells express both classical AR and ZIP9. It is therefore possible that the results summarized in Supplementary Figure S1 were due to the interactions of the peptides with the classical AR. In order to address the involvement of either ZIP9 or AR in the mineralization process induced by testosterone, Pep-1, or Pep-2, the mineralization experiments (Supplementary Figure S3) were repeated after silencing the expression of ZIP9 by siRNA. Control cells were treated with negative control siRNA (nc-siRNA). When cells were treated with nc-siRNA, Alizarin Red staining of calcium deposits was visible after four days in some cells (Figure 7A), but it was much stronger in cells that had been exposed to testosterone (Figure 7C), Pep-1 (Figure 7E), or Pep-2 (Figure 7G). When ZIP9 expression was silenced by ZIP9-specific siRNA, there was no longer any stimulation of Alizarin Red staining by testosterone (Figure 7D), Pep-1 (Figure 7F), or Pep-2 (Figure 7H), and the signal was at the same level as in control cultures treated with ZIP9-siRNA (Figure 7B) or nc-siRNA (Figure 7A).

After 11 days of incubation, cells that were treated with nc-siRNA responded to testosterone (Figure 7K), Pep-1 (Figure 7M), or Pep-2 (Figure 7O) with the highest amount Alizarin Red staining. When ZIP9 expression was suppressed by ZIP9-specific siRNA (Figure 7L,N,P), staining in the presence of testosterone or the peptides did not differ from that of controls that had been treated with either nc-siRNA (Figure 7I) or ZIP9-siRNA (Figure 7J). Quantification of the Alizarin Red staining (Figure 7Q,R) confirmed the results shown in the previous photomicrographs.

## 4. Discussion

The classical AR is not the sole receptor for testosterone or dihydrotestosterone. Recent investigations have established ZIP9 is as a membrane-bound androgen receptor of physiological, pathophysiological, pharmacological, and potentially clinical relevance. The androgen binding site of ZIP9 is localized on its extracellular surface and could therefore be selectively and specifically targeted, provided the availability of such probes. The investigation presented here is the result of the concerted application of bioinformatics, biochemistry, and cell biology with the goal of designing, producing, and investigating the effects of small peptides that target the extracellularly localized androgen binding site of ZIP9.

By re-calculating and applying the coordinates of a new and improved ZIP9 model as the basis for further calculations, six tetrapeptides capable of building complexes within the androgen binding site of ZIP9 characterized by the highest docking score, maximum surface contact area, minimum atomic contact energy, and negative FoldX energy were selected and then synthesized in order to address their interactions with ZIP9. ZIP9-mediated testosterone effects in two cell lines served as a litmus test for the actions of the peptides.

The ability of the peptides to bind within the androgen binding site of ZIP9 was addressed by determining their ability to displace T-BSA-FITC from the androgen binding site of ZIP9. All six peptides as well as testosterone were able to prevent the binding of the testosterone analog, which was consistent with the 3D model predicting the extracellular localization of the androgen binding site and which also validated the synthesized peptides as ZIP9 androgen binding site-specific probes. This latter conclusion was further verified by demonstrating the complete loss of T-BSA-FITC labeling from the L6 cell surface after silencing ZIP9 expression by siRNA. Testosterone stimulates muscle growth [46,47,48], and increased muscle mass helps to stabilize the skeleton and is therefore beneficial to patients suffering from osteopenia or osteoporosis [49,50,51]. Young as well as older men respond to testosterone with a dose-dependent increase in muscle fiber cross-sectional area and satellite cell number [52,53]. Myogenin is a muscle-specific member of the myo-D transcription factors that coordinates skeletal muscle development, myogenesis, and muscle repair [39]. Testosterone, whose administration increases skeletal muscle mass and strength [40,41], is a positive regulator of myogenin expression [54]. An essential phase of skeletal myogenesis is the fusion of mononucleated myoblasts to form multinucleated myotubes. A key player in this process is the focal adhesion kinase (FAK) [42,43]. The fact that the androgenic peptides designed in this study stimulate myogenin expression, FAK activation, and the formation of myotubes in myogenic cells implies that they might be beneficial in treating patients with bone diseases by increasing muscle mass. It is also conceivable that the myogenic activity of the peptides might benefit patients suffering from various myopathies. Thus, patients with hypogonadism who have a reduced production of testosterone often suffer from myotonic muscular dystrophy type 1 [55]. Patients affected by Duchenne’s muscular dystrophy respond positively to treatment with testosterone, although some side effects cannot be avoided [56].

The androgenic activity of the peptides was also demonstrated by their actions on the osteogenic cells SAOS-2. All peptides acted like testosterone by stimulating mineralization and by reducing alkaline phosphatase expression. Hypogonadism is one of the main causes of osteopenia and osteoporosis in older men. More than 30% of men aged older than 50 years are affected by these bone illnesses that can be diagnosed by measuring the activity of alkaline phosphatase. In men with osteoporosis or its precursor osteopenia, testosterone levels correlate in a reciprocal way with the expression of bone-specific alkaline phosphatase. Thus, at low testosterone levels, alkaline phosphatase expression is high and returns to normal levels after patients have undergone therapy with testosterone supplements [44]. At the same time, bone mineral density increases [44]. Alkaline phosphatase is an early marker of osteoblast differentiation and growth status [57,58]. Bone-specific alkaline phosphatase is currently one of the markers assessed in blood as a possible diagnostic for the onset of bone degradation and for the evaluation of the patient’s response to testosterone-enhancing therapies.

Taken together, the results in myogenic and osteoblastic cells lines imply that the testosterone-mimicking peptides presented in this investigation might be beneficial in the treatment of bone diseases in a twofold manner: by stimulating mineralization of the bone and by stimulating muscle mass production. The suitability of the peptides as a replacement for testosterone in the treatment of osteoporosis is currently being assessed and needs to be evaluated further. Nevertheless, these studies are at an early stage and various parameters such as intestinal uptake of the peptides or their stability or possible toxicity have to be assessed before they can be used clinically.

## 5. Conclusions

In the last two decades, great efforts have been made to develop and characterize selective androgen receptor modulators (SARMs) that can activate their receptor in a tissue-specific manner and promote the beneficial effects of androgens while avoiding unwanted side effects as much as possible [59]. Based on the results presented here, the peptides targeting the androgen binding site of ZIP9 could be viewed as being potential SARMs. Since their actions that stimulate the progression of myocytes to myotubes do not require the presence of the classical AR, it will be important to investigate their effects on muscle production and regeneration under experimentally induced hypogonadism. Multiple other uses of the androgenic peptides presented in this investigation are conceivable; however, at this stage we wish to emphasize that this is the first demonstration of the design and production of peptides that specifically target the androgen binding site of ZIP9 that induce testosterone-like effects of potentially substantial physiological significance.

## 6. Patents

The Justus-Liebig-University and the authors V.N.M., A.B., and G.S.-B. have submitted a patent application for the tetrapeptides: file number: EP 20209127.8; priority day: 23 November 2021.

## Figures and Tables

**Table 1:** Comparative analysis of three models generated using SWISS MODEL.

Template Structure	MolProbity Score	Clash Score	Ramachandran Favoured	Ramachandran Outliers	Rotamer Outliers	C-Beta Deviations	Bad Bonds	Bad Angles
5tsa.1.A	2.61	15.74	88.01	4.79	2.59	16	2/2204	67/2999
6pgi.1.A	2.48	14.66	90.03	2.75	2.16	9	0/2196	40/2988
mouse Zip9	2.1	0.45	85.5	5	10.9	18	0/2245	40/3054

**Table 2:** Identification of ZIP9-directed tetrapeptides and their properties. The programs and servers used for the identification of the peptides and their interactions with the androgen binding site of ZIP9 are presented in white letters.

PepComposer:						
Peptide Sequence	IAPG (Pep-1)	GVSG (Pep-2)	GVVG (Pep-3)	PQTG (Pep-4)	SGNL (Pep-5)	QAPG (Pep-6)
**FoldX server:**						
FoldX energy of ZIP9/tetrapeptide complexes (kcal/mol)	−2.2	−1.8	−1.2	−1.2	−0.8	−0.5
**PatchDock server:**						
Docking score	4718	4192	4082	4580	4744	4744
Surface contact area	556	495	461	538	589	524
Atomic contact energy (kJ/mol)	−232	−215	−166	−158	−201	−105
**AutodockVina:**						
Binding affinity of peptides for ZIP9 (kcal/mol)	−7.8	−6.3	−6.3	−6.9	−6.3	−7.7

**Table 3:** Coordination Residues (human ZIP9). A166 is the only amino acid that interacts with all compounds.

Testosterone	IAPG (Pep-1)	GVSG (Pep-2)	GVVG (Pep-3)	PQTG (Pep-4)	SGNL (Pep-5)	QAPG (Pep-6)
			A165			
A166	A166	A166	A166	A166	A166	A166
S170	S170	S170			S170	S170
				S173	S173	
	V174				V174	
						A181
		S230	S230	S230	S230	S230
		S232		S232	S232	
					S233	S233
V240	V240		V240			
M247	M247	M247				
L248	L248	L248				
				G244	G244	

## Data Availability

From the corresponding author, upon reasonable request.

## Supplementary Materials

The following are available online at https://www.mdpi.com/article/10.3390/biology11010019/s1, Figure S1: Scheme of tetrapeptide synthesis. Figure S2: Alizarin Red staining of osteoblastic SAOS-2 cells. Figure S3: Western blots used in Figure 3 of the manuscript.
