# Supplementary material for: The Effects of Tetrapeptides Designed to Fit the Androgen Binding Site of ZIP9 on Myogenic and Osteogenic Cells
Source: Biology (Basel). 2021 Dec 23;11(1):19. doi: 10.3390/biology11010019 (PMC8772937; doi:10.3390/biology11010019)
Supplement: Supplementary file 1 [file biology-11-00019-s001.zip › biology-1503020-supplementary/Figure1S.pptx]

## Slide 1
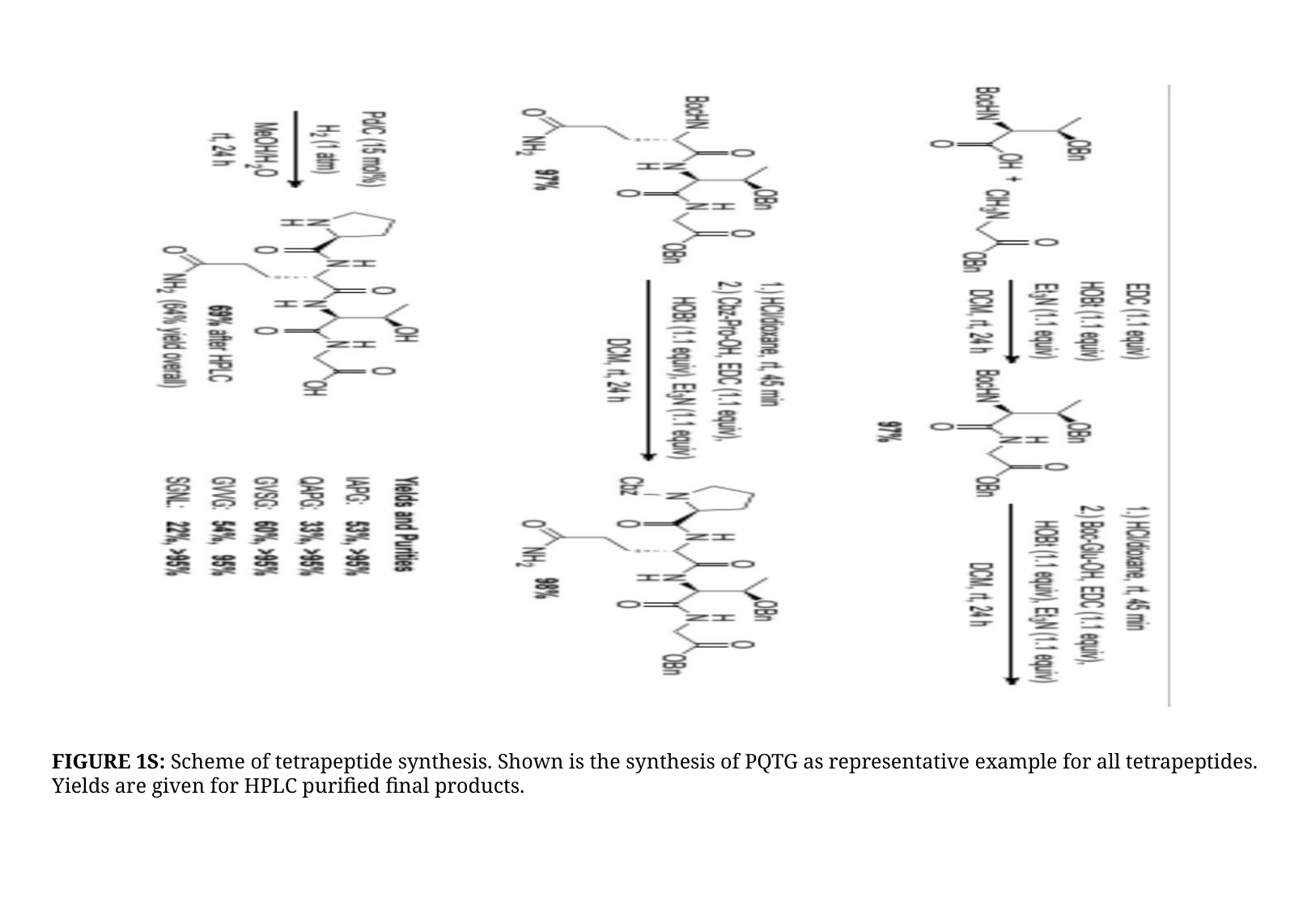

FIGURE 1S: Scheme of tetrapeptide synthesis. Shown is the synthesis of PQTG as representative example for all tetrapeptides.
Yields are given for HPLC purified final products.
