# Supplementary material for: The Effects of Tetrapeptides Designed to Fit the Androgen Binding Site of ZIP9 on Myogenic and Osteogenic Cells
Source: Biology (Basel). 2021 Dec 23;11(1):19. doi: 10.3390/biology11010019 (PMC8772937; doi:10.3390/biology11010019)
Supplement: Supplementary file 1 [file biology-11-00019-s001.zip › biology-1503020-supplementary/Figure2S.pptx]

## Slide 1
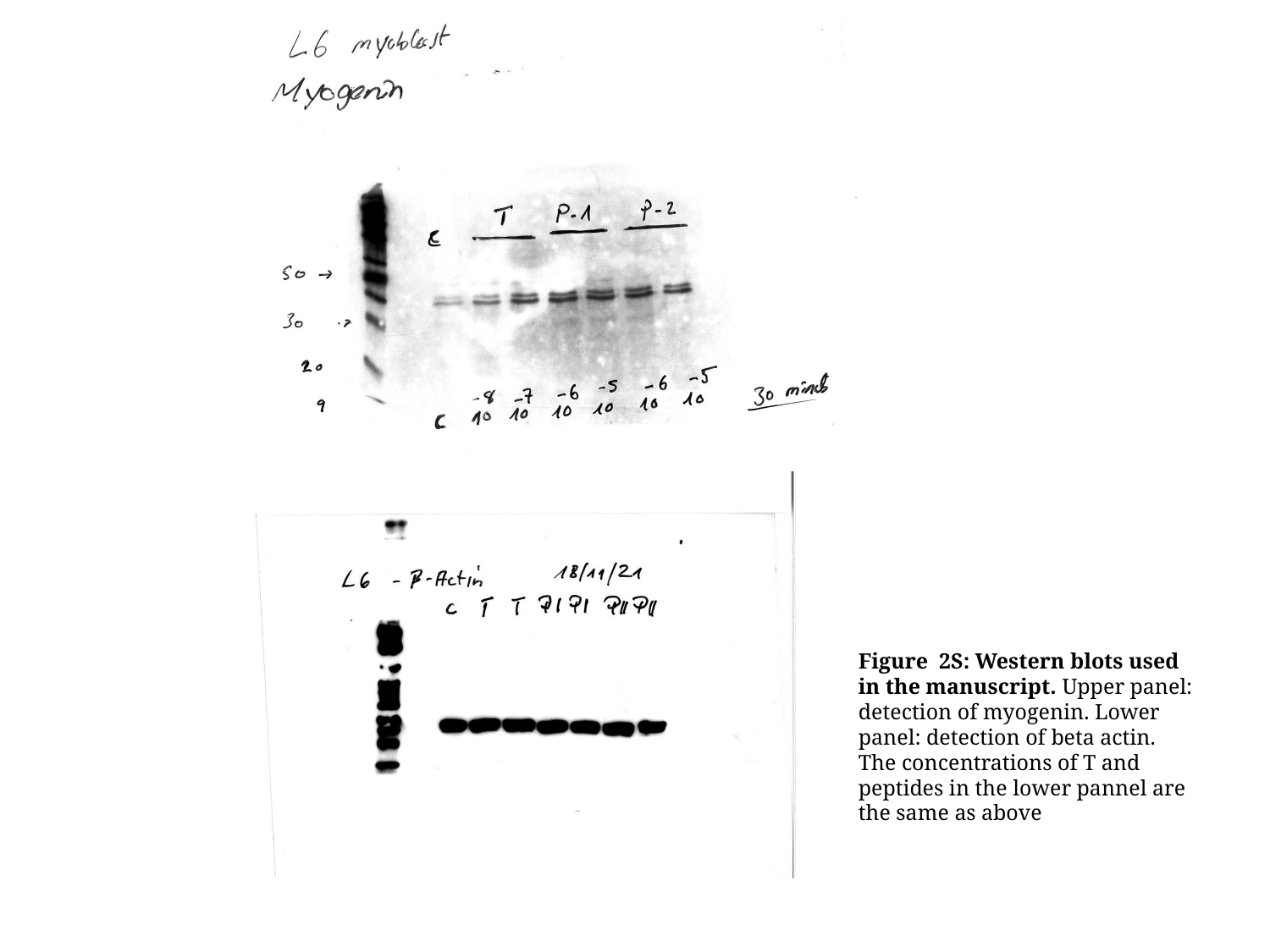

Figure 2S: Western blots used in the manuscript. Upper panel: detection of myogenin. Lower panel: detection of beta actin. The concentrations of T and peptides in the lower pannel are the same as above
