# Supplementary material for: The Effects of Tetrapeptides Designed to Fit the Androgen Binding Site of ZIP9 on Myogenic and Osteogenic Cells
Source: Biology (Basel). 2021 Dec 23;11(1):19. doi: 10.3390/biology11010019 (PMC8772937; doi:10.3390/biology11010019)
Supplement: Supplementary file 1 [file biology-11-00019-s001.zip › biology-1503020-supplementary/Figure3S.pptx]

## Slide 1
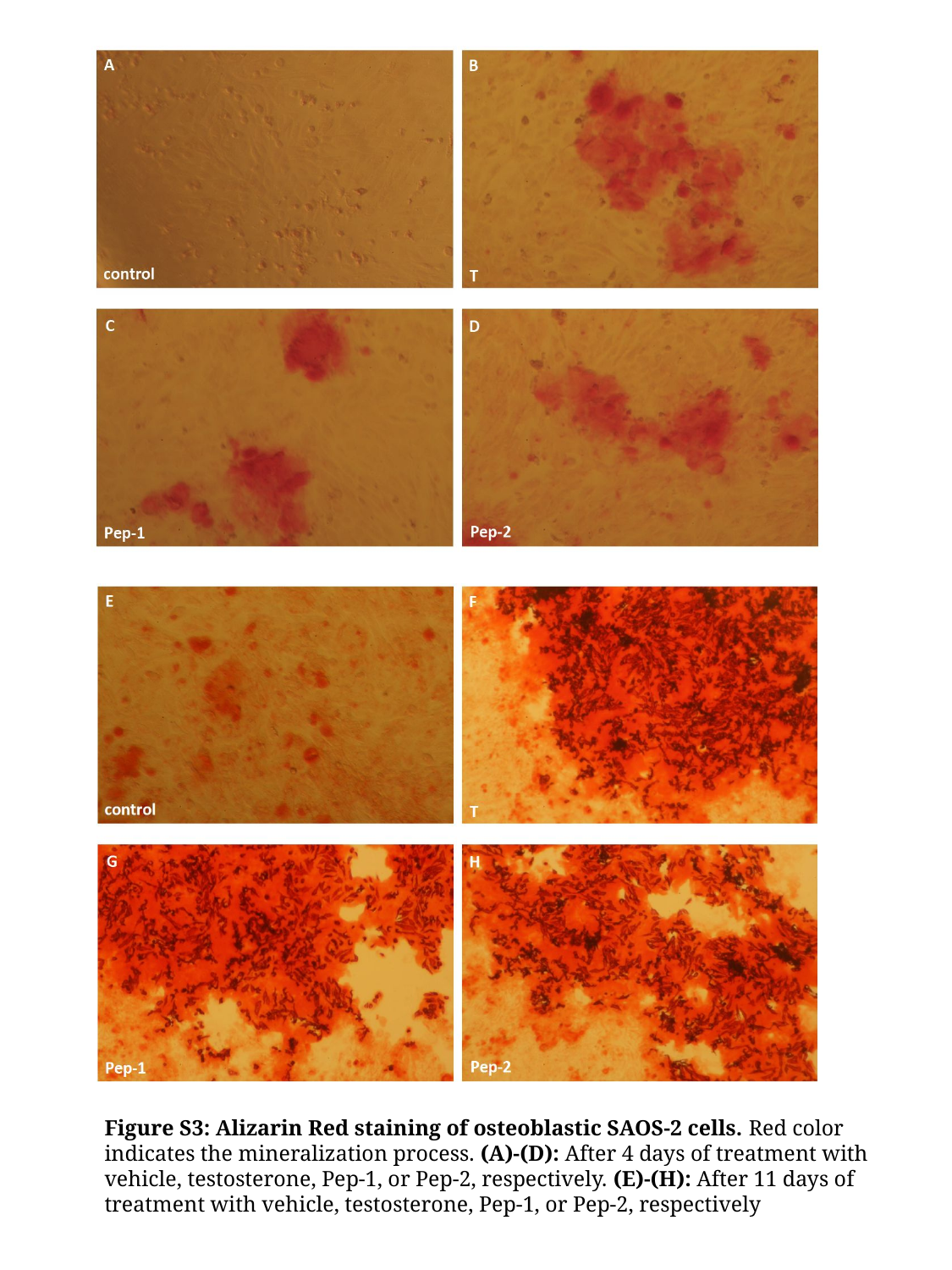

Figure S3: Alizarin Red staining of osteoblastic SAOS-2 cells. Red color indicates the mineralization process. (A)-(D): After 4 days of treatment with vehicle, testosterone, Pep-1, or Pep-2, respectively. (E)-(H): After 11 days of treatment with vehicle, testosterone, Pep-1, or Pep-2, respectively
